# The change in metabolic activity of a large benthic foraminifera as a function of light supply

**DOI:** 10.1038/s41598-023-35342-x

**Published:** 2023-05-22

**Authors:** Michael Lintner, Bianca Lintner, Michael Schagerl, Wolfgang Wanek, Petra Heinz

**Affiliations:** 1 Department of Palaeontology, University of Vienna, Vienna, Austria; 2 Department of Functional and Evolutionary Ecology, University of Vienna, Vienna, Austria; 3 Department of Microbiology and Ecosystem Science, University of Vienna, Vienna, Austria

**Keywords:** Ecosystem ecology, Microbial ecology, Marine biology

## Abstract

We studied metabolic activity of the symbiont-bearing large benthic foraminifer *Heterostegina depressa* under different light conditions. Besides the overall photosynthetic performance of the photosymbionts estimated by means of variable fluorescence, the isotope uptake (^13^C and ^15^N) of the specimens (= holobionts) was measured. *Heterostegina depressa* was either incubated in darkness over a period of 15 days or exposed to an 16:8 h light:dark cycle mimicking natural light conditions. We found photosynthetic performance to be highly related to light supply. The photosymbionts, however, survived prolonged darkness and could be reactivated after 15 days of darkness. The same pattern was found in the isotope uptake of the holobionts. Based on these results, we propose that ^13^C-carbonate and ^15^N-nitrate assimilation is mainly controlled by the photosymbionts, whereas ^15^N-ammonium and ^13^C-glucose utilization is regulated by both, the symbiont and the host cells.

## Introduction

### General introduction

Large benthic foraminifera (LBF) are essential components of shallow marine ecosystems like coral reefs and seagrass meadows. LBFs are sensitive to climate change and react almost immediately to changing physical parameters like temperature or salinity^1^. Recently, numerous studies have shown that foraminiferal communities are sensitive bioindicators for monitoring environmental parameters and their change: Schmidt et al.^2^ investigated the combined effects of warming and ocean acidification on LBFs, showing that elevated temperature had more negative effects on calcareous organisms than increased concentrations of dissolved CO_2_. This impact is, however, dependent on the examined taxa^3–5^. Especially those species which incorporate more Mg^2+^ into the carbonate tests are important for the chemical equilibrium in reefs as they have (post mortem) a high buffer capacity against daily pH fluctuations caused by community metabolism^6^. Generally, the assemblages of living LBFs are highly dependent on physical parameters like habitat depth, light supply and water motion^7^. Observing LBFs activity is suitable to detect chemical contaminants in seawater^8^. Thus, regular monitoring of LBFs can be used as an important tool to characterize the health state of coral reefs. This approach was first established by Hallock et al.^9^ as the FORAM index (Foraminifera in Reef Assessment and Monitoring) and is based on the changes in the foraminiferal assemblages associated with environmental changes. Following that, it is possible to classify the state of health of coral reefs by simply investigating the foraminiferal fauna composition^9, 10^.

### Light as key factor for LBFs

LBFs contribute a significant amount to the carbonate sediments worldwide due to their high abundance^11^. They also play a fundamental role in global carbon cycling and in sediment production in reefs^11^. Generally, LBFs contain photosymbionts which play a pivotal role in the development of large sizes up to several mm^12^. Species hosting photosymbionts have an increased test size, a special chamber arrangement and ultrastructural modifications to optimize the light supply within the foraminiferal organisms^13^. Especially in environments with elevated light supply paired with depleted dissolved organic matter content, algal symbionts are advantageous^14^. Interestingly, although most LBFs are mixotrophic, they are not able to survive for longer periods without their endosymbionts^15^. Not only a high diversity of eukaryotic algal symbionts are known for LBFs, but also cyanobacteria and bacteria are assumed to contribute significantly to the LBFs metabolism^16^. The endosymbionts are controlled by several factors like food availability of the host, water temperature, light supply and salinity^17^.

In this study we use *Heterostegina depressa* as a model, representing large rotalids, which host obligatory symbionts (diatoms). The adaption to different light intensities from this symbiosis is reflected by their morphological plasticity. At this point it should be mentioned, that also some miliolids are part of the LBFs. In contrast to the adaptive strategy to different light conditions from rotalids, miliolids establishing symbioses with a variety of algal symbionts^10^.

Pecheux^18^ measured test sizes of LBFs collected from different water depths (20–130 m) and found that their size is directly related (negative) to light supply. The importance of irradiance for symbiont bearing foraminifera is obvious and was already observed by earlier studies^19^. However also other factors might be significant for the abundance of LBFs: Nobes et al.^20^ found that irradiance flux only explained a small proportion of foraminifera distribution (based on the observation of large rotalids). Contrarily, the distance from the coast turned out to be the most important factor for LBF occurrence, whereby potentially also the nutrient flux will play a role in the foraminiferal distribution, but this aspect was not clarified by Nobes et al. In laboratory experiments, the same authors also found that the growth of the LBF *Heterostegina depressa* increased significantly at reduced light supply under continuous irradiance supply; therefore this taxon is considered a low light species. High irradiance of ~ 1200 µmol photons m^−2^ s^−1^ leads to increased mortality (50%) within a few weeks, whereas low light supply (60 µmol photons m^−2^ s^−1^) turned out as the light optimum for *H. depressa*^20^. These results fit to the findings of Röttger^21^, who postulated highest growth rates of *H. depressa* at low light supply. *H. depressa* is a species which is obligatorily dependent on the metabolic by-products of their symbionts and therefore shows a mixotrophic life style (= host cells are heterotrophic but obtain metabolites from their autotrophic symbionts) like other LBFs^22^. Because of the direct dependency on irradiance supply, this species is used for paleo-reconstruction of past water depths by analysing the occurrence of fossil LBFs^23^.

Though some studies^18, 20^ have been conducted on the growth and size distribution of LBFs related to irradiance supply, no study has dealt with nutrient uptake of LBFs as dependent on light supply to our knowledge. We assume that the utilization of certain carbon- and nitrogen-related compounds is conducted by the symbionts or is enhanced by their presence under light. However, other compounds, like dissolved organic material, will also be taken up and assimilated by the foraminifera itself or by osmosis where the symbionts are not involved. For that purpose, we measured nutrient uptake (nitrate, ammonium, carbonate and glucose) during prolonged darkness and compared it with foraminifera grown at a diurnal light cycle. In addition, pulse amplified modulated fluorescence analyses were conducted with an imaging fluorescence instrument to study potential effects of irradiance supply and prolonged darkness on symbiont performance.

With this study we want to clarify several aspects. First, it should be observed whether foraminifera absorb dissolved components of carbon and nitrogen in complete darkness. Based on this observation, further experiments with a normal daylight rhythm will be carried out to investigate the proportion of the up taken amount of elements by the symbionts. Finally, since LBFs are often used as model organisms, a statement should be obtained about which isotopes are best suited for further laboratory cultivation experiments.

These results contribute to a better understanding of the host-endosymbiont relationship between foraminifera and diatoms and clarify which nutrients are more likely to be taken up by the diatoms and which by the foraminifera itself. In addition, these results can also be used for paleontological studies. Since foraminiferal assemblages are often used as proxies for the reconstruction of paleoenvironments, light-factor experiments in particular provide new data on the distribution patterns of certain species.

## Material and methods

### Main culture

We used individuals of a permanent culture of *H. depressa*, hosted at the Department of Palaeontology at the University of Vienna. All selected foraminifera had a diameter of approximately 1250 µm. The main culture is maintained in an aquarium at 25 °C and 30 µmol photons m^−2^ s^−1^ photosynthetically active radiation (PhAR).

### Photosynthetic performance of the photobiont

**Figure 1:**
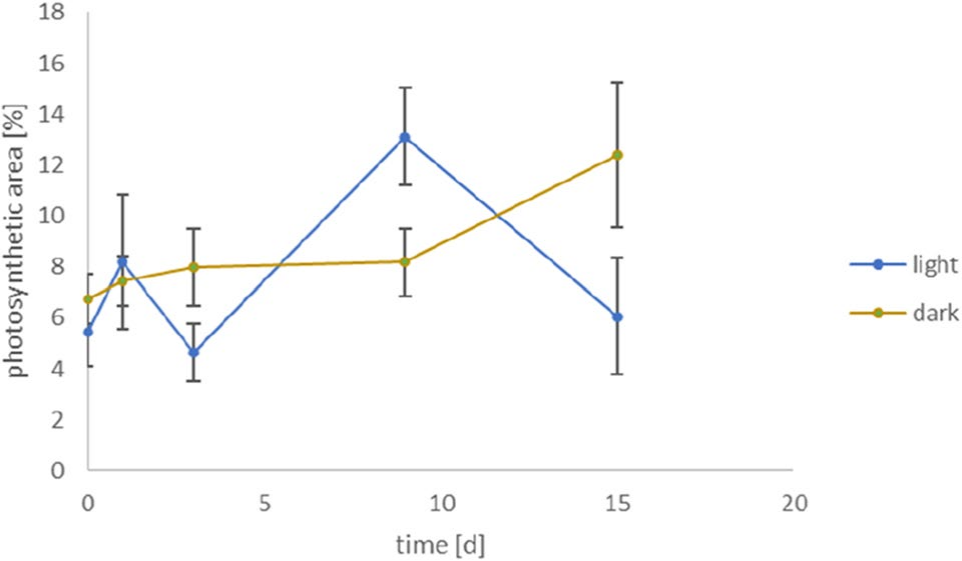
Photosynthetic mean area as a percentage of whole *H. depressa* specimens (n = 6, bars indicating standard error) grown under darkness or light:dark-cycle of 16:8 h.

Experiments were performed in six-well plates with placing a single individual in each well. The specimens were covered with 5 ml sterile filtered artificial seawater and were incubated at 25 °C. Six individuals were each incubated in total darkness or under a light:dark-cycle of 16:8 h at 30 µmol photons m^−2^ s^−1^, respectively (12 specimens in total). Photosynthetic performance of the photobiont symbionts of LBFs was measured several times during a period of 15 days using maximum variable chlorophyll fluorescence imaging of photosystem II (PSII; Imaging PAM Microscopy Version–Walz GmbH; excitation at 625 nm). Both, dark and light incubated foraminifera were measured at day 1, 3, 5 and 7 (Fig. 1). For this purpose, the same 12 individuals were measured every timepoint. The measured variable fluorescence as a proportion of maximum fluorescence yield (Fv/Fm) describes the difference between maximum fluorescence and minimum fluorescence (variable fluorescence), divided by maximum fluorescence, which is used as a measure of the maximum potential quantum efficiency of photosystem II^24^. Fv/Fm serves as a proxy for the integrity and physiological activity of the photosymbionts, ranging between 0.79 and 0.84, lower value indicating photobiont stress^24^. The PAM—images were evaluated using the software WinControl-3 (Walz GmbH); the photosynthetic area of each specimen was calculated with the software Image J (version 1.53 k, Java).

### Isotopic uptake experiments

Foraminifera were incubated for 1, 3 and 7 days in crystallisation dishes filled with 280 ml sterile filtered artificial seawater. Six foraminifera were placed into each dish, supplied separately with either isotopically enriched Na^15^NO_3_, ^15^NH_4_Cl, NaH^13^CO_3_ or ^13^C-glucose to a final concentration of 0.2 mM each. One set of foraminifera was incubated at a light: dark-cycle of 16:8 h at 30 µmol photons m^−2^ s^−1^, a second one in continuous darkness. In total 6 × 4 × 2 (number of replicates × isotopically enriched compounds × light conditions) foraminifera were incubated for this experiment. After the respective incubation times, the foraminifera were collected from each irradiance treatment and nutrient addition. For each treatment, 6 replicates were analysed individually. After incubation, the foraminifera were rinsed with distilled water and transferred to pre-weighted Sn capsules. The specimens were then dried for 3 days at room temperature and weighed. Then the tests were dissolved in 12.5 µl 2 M HCl and the organic remains dried at 50 °C for 3 days and reweighed. Measurements of the isotope ratios of each sample were carried out in the Stable Isotope Laboratory for Environmental Research (SILVER) of the University of Vienna. For a detailed description of the measurement and the calculation of the incorporated amount of isotopes, see Lintner et al.^25^.

### Statistics

The following hypothesis should be testes: different lighting conditions affect the activity of the symbionts (PAM experiments). Additionally, the hypothesis that the activity of the foraminifera is influenced by different chemical nitrogen or carbon sources components will also be investigated (isotopic uptake experiments). For statistical analysis, repeated measurement one-way ANOVA (level of significance = 95%) was performed for the PAM experiments over time to test if, prolonged darkness significantly altered the overall photosynthetic performance of the photobiont compared to natural irradiance supply. Two-way ANOVAs were used for the isotopic uptake to test if light supply and time affected the uptake of enriched ^13^C- and ^15^N-compounds. We used the software Past 4.03 and set the level of significance to 95%.

## Results

### Performance of the photosymbionts

The results of the PAM observations after experimental start and after day 1, 3, 7 and 15 are shown in Fig. 1 (values are provided in the supplementary file). During the whole experiment, Fv/Fm of all individuals was in the range between 0.6 and 0.8, which indicates a healthy state of the photosymbionts. Two-way ANOVA of the photosynthetic area over a period of 15 days, between the dark- and light-incubated foraminifera show a significantly difference between the light cycle (*p* = 0.027) and time (*p* < 0.001) and also their interaction (*p* < 0.001).

Within the dark incubated foraminifera, we observed no significant change in photosynthetic area over 7 days (rm-ANOVA, *p* = 0.110). Just a significant increase (rm-ANOVA, *p* < 0.001) of the photosynthetic area was observes from day 7 to 15.

### Isotopic uptake experiments

The rate of isotope incorporation differed significantly (one-way ANOVA) depending on the type of offered carbon form (carbonate > glucose, *p* < 0.001) and nitrogen form (nitrate > ammonium, *p* < 0.001). Two-way ANOVA (cycle × time) was performed to see if there are differences in the uptake of isotopes during light exposure and over time. Natural light supply in contrast to complete darkness, highly significantly increased the uptake of carbonate, nitrate and ammonium (*p* < 0.001) and significantly for glucose (*p* = 0.048). The interaction between cycle and time was significant for glucose (*p* = 0.020), carbonate (*p* < 0.001) and nitrate (*p* < 0.001), but not for ammonium (*p* = 0.164). Tracer uptake increased with time (Table 1) for all compounds under light conditions, except ammonium. Under dark conditions tracer uptake only increased for carbonate and ammonium, but not for glucose (*p* = 0.087) and nitrate (*p* = 0.376) (Table 1).

**Table 1:** One-way ANOVA of the isotopic uptake with time (n = 6, *Df* = 2, significant *p* values are in bold).

Compound	Element	Exposure	Sum of sq	F value	*p* value
Glucose	13C	Light	0.000651	14.93	**< 0.001**
	13C	Dark	0.000104	2.894	0.087
Carbonate	13C	Light	0.009454	12.51	**0.001**
	13C	Dark	0.000000	6.946	**0.007**
Nitrate	15N	Light	0.006748	46.26	**< 0.001**
	15N	Dark	0.000000	1.044	0.376
Ammonium	15N	Light	0.000216	1.81	0.198
	15N	Dark	0.000000	4.989	**0.022**

**Figure 2:**
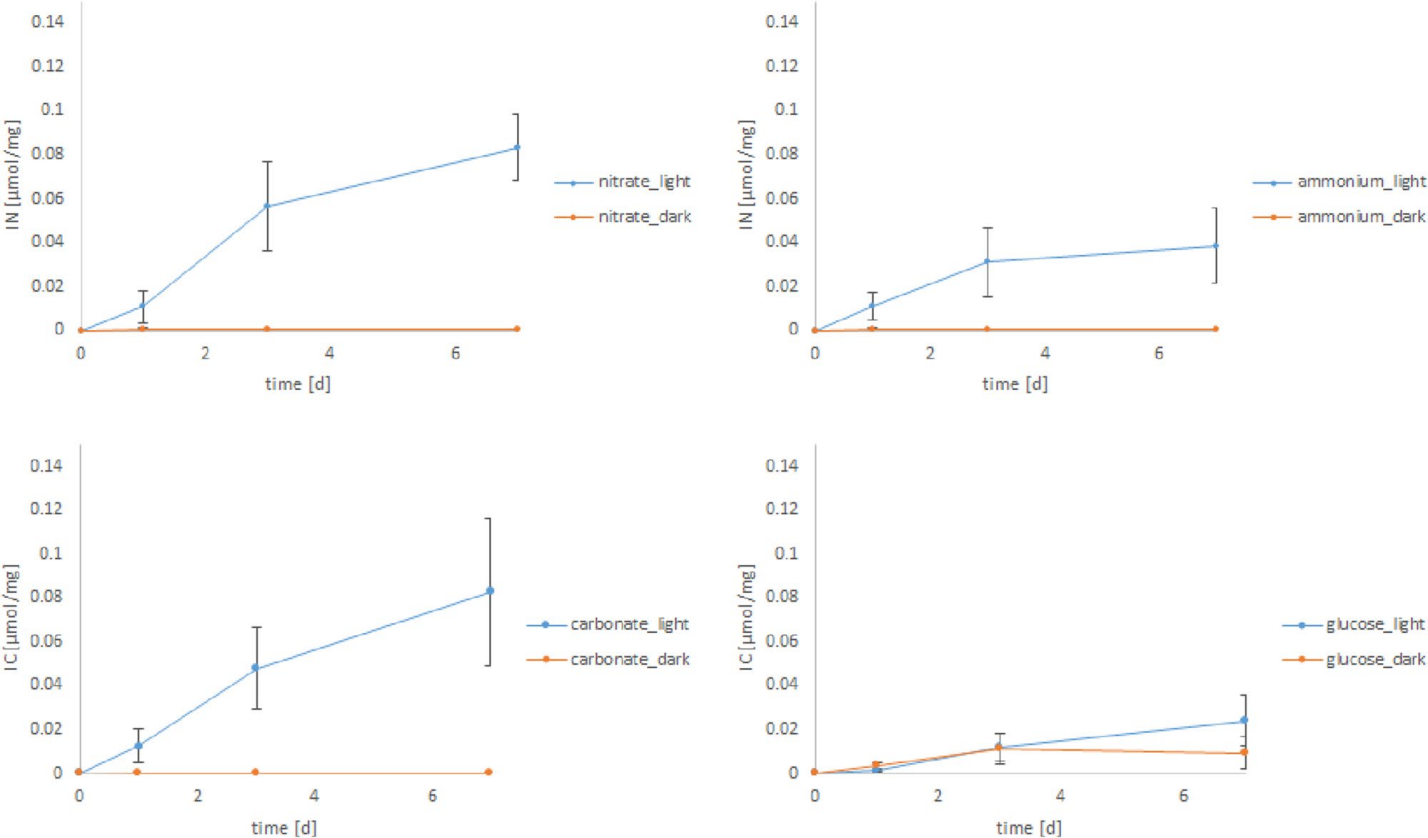
Incorporated amount of isotopes during dark and light experiments over 7 days. The blue line represents the amount of up taken N or C compound at light exposure (16:8 h light:dark), the orange line the element uptake in complete darkness. The mean of 6 individuals was used for each data point. Bars indicating standard error.

For nitrate, ammonium and carbonate, the element uptake during darkness was negligible (Fig. 2). Nitrate and carbonate uptake were higher than that of ammonium and glucose under natural light conditions (16:8 h light:dark). The uptake of nitrate and carbonate in the light was approximately twice compared to ammonium and glucose, respectively. In prolonged darkness, a substantial uptake of tracer was only recorded for glucose.

## Discussion

*Heterostegina depressa* is known as a low light species (oligophotic), i.e. well-adapted to grow under low light conditions^20^. We found that the photosynthetic area of the foraminiferal symbionts remained constant over 7 days of continuous darkness and show a slightly increase between 7 and 15 days (Fig. 1). This means that even after 15 days without light, the photosymbionts of *H. depressa* were alive and adapt to these conditions. Interestingly, there was no uptake and assimilation of carbonate and inorganic nitrogen during this time (Fig. 2).

Past experiments with dissolved carbonate show that LBFs can take it up by diffusion^26^. This uptake then follows a linear increase in the C concentration in the cytoplasm of the foraminifera as a function of time. However, we were only able to record a linear increase of the C concentration in the foraminifera during the experiments, which were carried out under light exposure. The dark incubated foraminifera show no uptake of carbon, which suggests that the C uptake does not take place by diffusion but by enzymatic activity as already suspected by Ter Kuile et al.^26^.

During prolonged darkness, foraminifera operate purely heterotrophic, as shown by the uptake of dissolved glucose, which was likely metabolized for energy generation in the absence of any transfer of photosynthates and other metabolites from the photosymbionts. Glucose uptake might also be promoted by the presence of bacteria, since some foraminifera also contain heterotrophic bacteria as symbionts, which can quickly digest glucose^12^. Another explanation could be an active uptake and digestion of enriched bacteria—its presence cannot be ruled out during an experiment for more than 3 days. Röttger et al.^27^ reported, that *H. depressa* can active feed on algae, but this food uptake just play a minor role in the energy budget of the foraminifera. It can therefore also be hypothesized that the uptake of glucose is caused indirectly by the phagocytosis of bacteria that have previously enriched themselves with ^13^C. The bacteria uptake and the so called “bacteria farming” is a widely known strategy of small benthic foraminifera^28^. At the moment, this feeding strategy was only observed from non-symbiont bearing foraminifera. However, it cannot be ruled out that bacteria settle on the surface of the foraminifera, which then metabolize glucose. The ^13^C-enriched metabolites of the bacteria can then be released into the culture water and absorbed through close contact by the foraminifera. In order to understand this more closely, further studies using TEM or NanoSIMS must be carried out. These studies would also clarify whether this species is able to uptake glucose via osmotrophy.

The isotope incorporation increased with time under natural light conditions (16:8 h light: dark). There was the same rising pattern for nitrate, ammonium, carbonate and glucose incorporation, which was fundamentally different from the pattern under continuous darkness. Although glucose uptake was similar, nitrate, ammonium, and carbonate uptake increased under irradiances supply substantially already after 7 days. We assume that glucose uptake is mostly driven by the heterotrophic foraminiferal host cell or by bacterial symbionts^29^ and used to generate energy and carbon skeletons for physiological processes. Under light supply, additional energy and organic compounds for foraminiferal growth are generated by the photobionts. Lintner et al.^25^ investigated the element uptake of the obligatory heterotrophic *Cribroelphidium selseyense*. During the first 7 days, they found only a marginal assimilation of carbonate and nitrate^25^, which however increased afterwards probably because of symbiotic bacterial activity. At the same time, *C. selseyense* showed a continuous uptake of ammonium during the whole experiment^25^. We conclude from our data, that assimilation of carbonate, nitrate and ammonium are light-dependent and triggered by the activity of the phototobionts, while glucose uptake is continued in darkness thus maintaining the holobionts metabolism. This helps the foraminifera to survive prolonged dark phases.

We studied nitrogen using the two inorganic compounds ammonium and nitrate and were able to prove a much higher nitrate uptake (Fig. 2). For both, nitrate and ammonium, there was no uptake in the dark, which implies that inorganic nitrogen assimilation was performed by only the photosymbionts, mediated under light supply. Interestingly, during the experiments, which were carried out completely in the dark, no uptake of nitrate was recorded (see Fig. 2). From studies with marine diatoms, however, it is known that diatoms accumulate nitrate in the cells during darkness^30^. This now suggests that the foraminifera itself is not active even in the dark, nor does it have osmotrophy during this period, allowing dissolved nitrate to be carried to the symbionts. Such behaviour could be compared to dormancy in foraminifera^31^. Dormancy can be caused by exogenous factors such as stressful environmental conditions (here lack of light during dark conditions) and leads to a strong reduction in metabolism. This hypothesis can now be reconciled very well with our results. It now appears that the here investigated foraminifera goes into a kind of dormancy during complete darkness and reduced the metabolism to an absolute minimum. However, since there is no uptake of any isotope during total darkness, this strategy applies not only to the foraminifera but also to their photosymbionts.

In general, both inorganic nitrogen forms (nitrate and ammonium) can be used by photoautotrophs (algae and higher plants) as nitrogen source^32^. For metabolic pathways (amino acids, proteins, nucleic acids and else), both inorganic nitrogen forms first need to be incorporated into amino acids, which in the case of nitrate requires additional reduction equivalents, energy and enzymatic reactions^33^. For many photoautotrophic organisms a mixture of both compounds led to the highest nitrogen uptake in plants^30^. Kronzucker et al.^32^ reported that nitrate uptake and assimilation is inhibited at high ammonium concentrations. This aspect can be excluded for our results since we incubated the foraminifera separately with nitrate and ammonium. Further, Dortch^34^ postulated that the preferred nitrogen source of phytoplankton is ammonium, which does not fit to our results. These differences can probably be explained by the positive effect of nitrate uptake on the cation–anion balance of phototrophic organisms (phytoplankton), allowing higher nitrogen uptake and growth rates with nitrate than with ammonium^33^.

In the past, some cultivation experiments were carried out with foraminifera, which had either light or temperature as a stress factor^35^. However, the temperature effect on LBFs is species specific and it has been shown that temperatures above 31 °C lead to a rapid death of the photosymbionts in *H. depressa*^36^. Since the light condition was constant in the experiments by Schmidt et al.^36^ and the temperature in our experiments, it cannot be stated which parameter has a stronger effect on the foraminifera. In order to examine this aspect more closely, cross-design experiments with 2 variables (temperature × light supply) must be carried out in the future.

It was shown that the availability of light is the essential factor for the distribution of foraminifera with depth^35^. Presumably not only the daylight but also the moonlight plays a role here. Observations showed that LBFs grown in the natural environment have oscillations in their chamber volume, which is probably caused by lunar and tidal cycles^37^. It is assumed that the lunar cycle influences the productivity of the photosymbionts in LBFs and thus has a positive effect on the activity of the symbionts at full moon night^37^. However, the light intensity of moonlight is much lower than that of sunlight, only about 0.0024 μmol m^−2^ s^−1^^38^ which is around 12.5 k times lower than that in our experiment. It should be noted, that in sunlight all visible wavelengths are relatively equally present, whereas in moonlight the wavelengths are generally cantered around 400 nm^38^. If this wavelength-dependent irradiation affects the metabolism of *H. depressa* or not has not yet been investigated and could certainly shed more light on whether moonlight has an effect on the LBF symbionts.

Laboratory experiments have shown that *H. depressa* is a low light species^21^ and can therefore survive even in very poor light conditions. However, based on the results of our study, it can be clearly shown that in complete darkness the foraminifera do not absorb any essential nutrients. Recent studies have even shown that sequestered chloroplasts in foraminifera degrade within a few days when exposed to high light conditions and also have a photobleaching effect^39^. Even foraminifera, which have neither photosymbionts nor sequestered chloroplasts, can cope better with less light than with high light intensities^40^. All of these results and the data from this study suggest that high light intensities has a significant negative effect on their metabolism, but light is an essential factor for foraminifera with photosymbionts to survive.

## Conclusion

The uptake of carbonate, nitrate, ammonium and glucose in *H. depressa* is highly dependent on the availability of light. Under dark conditions, the organisms take up mainly glucose to provide energy for maintaining the metabolic processes. If foraminifera are exposed to light, the photosymbionts are primarily responsible for uptake and assimilation of carbonate, nitrate and ammonium. Based on these results, in future uptake experiments with *H. depressa* it is recommended to enrich the culture water with carbonate and ammonium nitrate in order to offer best conditions to study the activity of the foraminifera and their symbionts with changing environmental conditions.

## Supplementary Information

Supplementary Information.

## Data Availability

The datasets used and/or analysed during the current study available from the corresponding author on reasonable request.
