# Author Correction: The change in metabolic activity of a large benthic foraminifera as a function of light supply

**DOI:** 10.1038/s41598-023-36353-4

**Published:** 2023-06-06

**Authors:** Michael Lintner, Bianca Lintner, Michael Schagerl, Wolfgang Wanek, Petra Heinz

**Affiliations:** 1 Department of Palaeontology, University of Vienna, Vienna, Austria; 2 Department of Functional and Evolutionary Ecology, University of Vienna, Vienna, Austria; 3 Department of Microbiology and Ecosystem Science, University of Vienna, Vienna, Austria

Correction to: *Scientific Reports* 10.1038/s41598-023-35342-x, published online 22 May 2023

In the original version of this Article, Michael Lintner was incorrectly listed as a corresponding author. The correct corresponding author for this Article is Petra Heinz. Correspondence and request for materials should be addressed to petra.heinz@univie.ac.at.

The original Article has been corrected.

