# Supplementary material for: The change in metabolic activity of a large benthic foraminifera as a function of light supply
Source: Sci Rep. 2023 May 22;13:8240. doi: 10.1038/s41598-023-35342-x (PMC10203359; doi:10.1038/s41598-023-35342-x)
Supplement: Supplementary file 1 — Supplementary Information. [file 41598_2023_35342_MOESM1_ESM.pdf]

Data from the PAM-experiments:

D and L stands for the light cycles dark (D) and exposes 16:8h light:dark (L). The D and L values numbered in the first column refer to the 6 replicates for each parameter. Time is given in days and area means the photosynthetic active area.

	cycle	time	area
D1	D	0	8.423
D2	D	0	6.146
D3	D	0	7.23
D4	D	0	5.987
D5	D	0	6.901
D6	D	0	5.887
L1	L	0	4.256
L2	L	0	5.083
L3	L	0	6.023
L4	L	0	7.867
L5	L	0	5.021
L6	L	0	4.411
D1	D	1	7.867
D2	D	1	8.012
D3	D	1	7.765
D4	D	1	8.421
D5	D	1	5.765
D6	D	1	6.911
L1	L	1	8.741
L2	L	1	10.955
L3	L	1	8.634
L4	L	1	10.695
L5	L	1	4.666
L6	L	1	5.395
D1	D	2	8.912
D2	D	2	5.435
D3	D	2	7.986
D4	D	2	7.719
D5	D	2	9.945
D6	D	2	8.012
L1	L	2	5.638
L2	L	2	5.849
L3	L	2	4.423
L4	L	2	5.318
L5	L	2	3.026
L6	L	2	3.72
D1	D	9	9.686
D2	D	9	6.48
D3	D	9	8.789
D4	D	9	7.991
D5	D	9	9.341
D6	D	9	6.767
L1	L	9	10.175
L2	L	9	13.069
L3	L	9	12.678
L4	L	9	12.776
L5	L	9	13.855
L6	L	9	16.058
D1	D	15	12.755
D2	D	15	10.23
D3	D	15	9.47
D4	D	15	15.649
D5	D	15	10.34
D6	D	15	15.962
L1	L	15	4.15
L2	L	15	8.69
L3	L	15	5.013
L4	L	15	3.176
L5	L	15	8.174
L6	L	15	7.19

	cycle	time	Fv/Fm
D1	D	0	0.635
D2	D	0	0.685
D3	D	0	0.738
D4	D	0	0.836
D5	D	0	0.664
D6	D	0	0.736
L1	L	0	0.719
L2	L	0	0.811
L3	L	0	0.627
L4	L	0	0.678
L5	L	0	0.633
L6	L	0	0.647
D1	D	1	0.745
D2	D	1	0.712
D3	D	1	0.699
D4	D	1	0.81
D5	D	1	0.732
D6	D	1	0.671
L1	L	1	0.681
L2	L	1	0.833
L3	L	1	0.746
L4	L	1	0.615
L5	L	1	0.6
L6	L	1	0.674
D1	D	2	0.751
D2	D	2	0.864
D3	D	2	0.613
D4	D	2	0.798
D5	D	2	0.745
D6	D	2	0.768
L1	L	2	0.644
L2	L	2	0.719
L3	L	2	0.693
L4	L	2	0.777
L5	L	2	0.81
L6	L	2	0.682
D1	D	9	0.634
D2	D	9	0.766
D3	D	9	0.691
D4	D	9	0.823
D5	D	9	0.603
D6	D	9	0.798
L1	L	9	0.734
L2	L	9	0.765
L3	L	9	0.615
L4	L	9	0.654
L5	L	9	0.625
L6	L	9	0.765
D1	D	15	0.846
D2	D	15	0.635
D3	D	15	0.621
D4	D	15	0.625
D5	D	15	0.655
D6	D	15	0.634
L1	L	15	0.641
L2	L	15	0.671
L3	L	15	0.619
L4	L	15	0.627
L5	L	15	0.666
L6	L	15	0.652

Data from the isotopic uptake experiments:

The light cycle here describes an illumination of 16:8h (light:dark) and the dark cycle corresponds to permanent darkness. The name given was the isotope (^{13}C for glucose and carbonate, ^{15}N for nitrate and ammonium) that was added separately to the culture water. IC and IN describe the incorporated amount of C or N depending on which element was incubated with.

cycle	name	time	IC/N
light	glucose	1	0.00078693
light	glucose	1	0.00228566
light	glucose	1	0.00299097
light	glucose	1	0.00131
light	glucose	1	0.00050851
light	glucose	1	0.00170993
light	glucose	3	0.0063002
light	glucose	3	0.01793201
light	glucose	3	0.01690229
light	glucose	3	0.00486471
light	glucose	3	0.01817673
light	glucose	3	0.01311692
light	glucose	6	0.00992043
light	glucose	6	0.01391129
light	glucose	6	0.03479929
light	glucose	6	0.01890805
light	glucose	6	0.02678787
light	glucose	6	0.03010693
light	carbonat	1	0.00354999
light	carbonat	1	0.01457141
light	carbonat	1	0.02724217
light	carbonat	1	0.00438558
light	carbonat	1	0.004129
light	carbonat	1	0.00948974
light	carbonat	3	0.06055186
light	carbonat	3	0.02904247
light	carbonat	3	0.060192
light	carbonat	3	0.08122081
light	carbonat	3	0.04797228
light	carbonat	3	0.03119769
light	carbonat	6	0.16179867
light	carbonat	6	0.11335886
light	carbonat	6	0.08254987
light	carbonat	6	0.05250018
light	carbonat	6	0.08046167
light	carbonat	6	0.04893742
light	nitrate	1	0.00262488
light	nitrate	1	0.00391494
light	nitrate	1	0.00236403
light	nitrate	1	0.00708143
light	nitrate	1	0.00891983
light	nitrate	1	0.02102879
light	nitrate	3	0.03466337
light	nitrate	3	0.05087823
light	nitrate	3	0.0690483
light	nitrate	3	0.07920106
light	nitrate	3	0.04802166
light	nitrate	3	0.03753561
light	nitrate	6	0.06760026
light	nitrate	6	0.06736094
light	nitrate	6	0.06123462
light	nitrate	6	0.08270752
light	nitrate	6	0.07781018
light	nitrate	6	0.08167244
light	ammonium	1	0.01132176
light	ammonium	1	0.01195932
light	ammonium	1	0.01416596
light	ammonium	1	0.0021358
light	ammonium	1	0.02221561
light	ammonium	1	0.00344289
light	ammonium	3	0.00981851
light	ammonium	3	0.01065459
light	ammonium	3	0.01299373
light	ammonium	3	0.05047159
light	ammonium	3	0.00917595
light	ammonium	3	0.01086155
light	ammonium	6	0.02047764
light	ammonium	6	0.02632824
light	ammonium	6	0.02853327
light	ammonium	6	0.02729246
light	ammonium	6	0.02289338
light	ammonium	6	0.01170181

dark	glucose	1	0.00472319
dark	glucose	1	0.00061656
dark	glucose	1	0.00383798
dark	glucose	1	0.00391284
dark	glucose	1	0.00524459
dark	glucose	1	0.00127173
dark	glucose	3	0.00699672
dark	glucose	3	0.0201005
dark	glucose	3	0.01526063
dark	glucose	3	0.01359157
dark	glucose	3	0.01083971
dark	glucose	3	0.00061625
dark	glucose	6	0.0039298
dark	glucose	6	0.02359242
dark	glucose	6	0.00569693
dark	glucose	6	0.01258225
dark	glucose	6	0.00432762
dark	glucose	6	0.00615108
dark	carbonat	1	0.00023292
dark	carbonat	1	0.00014155
dark	carbonat	1	0.00015794
dark	carbonat	1	0.0001422
dark	carbonat	1	0.00013612
dark	carbonat	1	0.00014989
dark	carbonat	3	0.00011153
dark	carbonat	3	5.8661E-05
dark	carbonat	3	0.00016689
dark	carbonat	3	8.5048E-05
dark	carbonat	3	4.256E-05
dark	carbonat	3	0.00014602
dark	carbonat	6	0.00018744
dark	carbonat	6	0.00021951
dark	carbonat	6	0.00046628
dark	carbonat	6	0.00018148
dark	carbonat	6	0.00020391
dark	carbonat	6	0.00027035
dark	nitrate	1	0.00038035
dark	nitrate	1	0.00065314
dark	nitrate	1	0.00029829
dark	nitrate	1	0.00044324
dark	nitrate	1	0.00046496
dark	nitrate	1	0.00177895
dark	nitrate	3	0.0006231
dark	nitrate	3	0.0004086
dark	nitrate	3	0.00032346
dark	nitrate	3	0.00023427
dark	nitrate	3	0.00035615
dark	nitrate	3	0.00027757
dark	nitrate	6	0.00035863
dark	nitrate	6	0.00095997
dark	nitrate	6	0.00064245
dark	nitrate	6	0.00030362
dark	nitrate	6	0.00060129
dark	nitrate	6	0.00038495
dark	ammonium	1	0.00028753
dark	ammonium	1	0.00047354
dark	ammonium	1	0.00062827
dark	ammonium	1	0.00070381
dark	ammonium	1	0.0013071
dark	ammonium	1	0.00042419
dark	ammonium	3	0.00047545
dark	ammonium	3	0.00017469
dark	ammonium	3	7.9613E-05
dark	ammonium	3	0.0003155
dark	ammonium	3	0.00011878
dark	ammonium	3	8.6142E-05
dark	ammonium	6	0.00026521
dark	ammonium	6	0.0002912
dark	ammonium	6	9.3753E-05
dark	ammonium	6	0.00011087
dark	ammonium	6	0.00060205
dark	ammonium	6	0.00035111
